# Efficient and Stable Perovskite Solar Cells and Modules Enabled by Tailoring Additive Distribution According to the Film Growth Dynamics

**DOI:** 10.1007/s40820-024-01538-7

**Published:** 2024-10-15

**Authors:** Mengen Ma, Cuiling Zhang, Yujiao Ma, Weile Li, Yao Wang, Shaohang Wu, Chong Liu, Yaohua Mai

**Affiliations:** 1 Institute of New Energy Technology, College of Physics & Optoelectronic Engineering, Jinan University, Guangzhou, 510632 People’s Republic of China; 2 Key Laboratory of New Semiconductors and Devices of Guangdong Higher Education Institutes, Jinan University, Guangzhou, 510632 People’s Republic of China; 3 Guangdong Mellow Energy Co., Limited, Yuanming Road, Zhuhai, 519075 Guangdong People’s Republic of China

**Keywords:** Gas quenching, Additive distribution, Buried passivation, Blade coating, Crystallization dynamics

## Abstract

Gas quenching and vacuum quenching process are widely applied to accelerate solvent volatilization to induce nucleation of perovskites in blade-coating method. In this work, we found these two pre-crystallization processes lead to different order of crystallization dynamics within the perovskite thin film, resulting in the differences of additive distribution. We then tailor-designed an additive molecule named 1,3-bis(4-methoxyphenyl)thiourea to obtain films with fewer defects and holes at the buried interface, and prepared perovskite solar cells with a certified efficiency of 23.75%. Furthermore, this work also demonstrates an efficiency of 20.18% for the large-area perovskite solar module (PSM) with an aperture area of 60.84 cm^2^. The PSM possesses remarkable continuous operation stability for maximum power point tracking of T_90_ > 1000 h in ambient air.

**Supplementary Information:**

The online version contains supplementary material available at 10.1007/s40820-024-01538-7.

## Highlights

- Two pre-crystallization processes of gas quenching and vacuum quenching lead to different order of crystallization dynamics within the perovskite thin film, resulting in the differences of additive distribution.
- A tailor designed 1,3-bis(4-methoxyphenyl)thiourea was utilized to improve the buried interface, leading to a certified efficiency of 23.75% for blade-coated perovskite solar cell.
- The perovskite solar module (aperture area: 60.84 cm^2^) demonstrates an efficiency of 20.18% with excellent operational stability (maximum power point tracking of T_90_ > 1000 h).

**Supplementary Information:**

The online version contains supplementary material available at 10.1007/s40820-024-01538-7.

## Graphical abstract

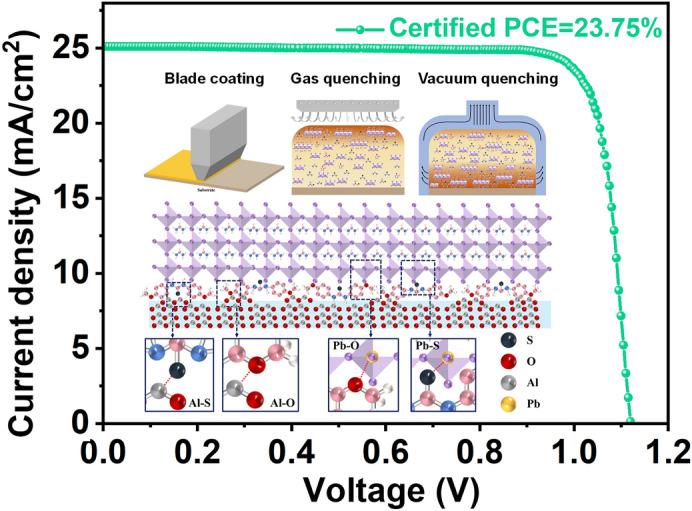

## Introduction

The efficiency of perovskite solar cells (PSCs) has continued to grow rapidly, as the small-area laboratory PSCs manufactured by the solution method have gained the certified power conversion efficiency (PCE) up to 26.7% [1]. The challenge to achieve high-quality perovskite thin films via solution method can be associated to the nucleation process that taken place within seconds and overlap each other, making the dynamics control needs to be strict, especially for the large-area perovskite thin film [2–4]. In general, rapid nucleation for pre-crystallization followed with slow crystal growth is necessary to form high-quality perovskites with fully surface coverage and large grain size [5, 6]. Anti-solvent extraction provides a picky strategy to obtain a perfect intermediate phase in spin-coating process that provides seed crystals for the growth of compact polycrystalline thin film with low bulk defects [7–9]. The anti-solvent method is not conducive to triggering uniform nucleation on perovskite surfaces with an area greater than 50 cm^2^, and it is difficult to obtain smooth perovskite films with high surface coverage [10–14]. Similarly, in the blade coating, gas quenching (GQ) and/or vacuum quenching (VQ) process are applied to accelerate solvent volatilization [15–18]. However, the concentration of wet film is relatively low before the quenching carried out, resulting in the solvent volatilization and reaching the minimum concentration for nucleation will take more times than the anti-solvent method. Ternes et al. obtained supersaturation rates of common quenching methods at critical concentrations by modeling, yielding ~ 10^2^ s^−1^ for anti-solvent quenching, ~ 10^−3^ − 10^−1^ s^−1^ for VQ, and ~ 10^−5^ − 10^0^ s^−1^ for GQ [19]. Low nucleation rate leads to inferior crystal, and this may be the reason why the efficiency of blade-coated PSCs still far leg behind the spin-coated counterparts.

The inference above prompted us to investigate the difference between GQ and VQ processes, because the different extraction processes seriously affect the crystallization dynamics, which is the key factor to determine the as-prepared film quality. For GQ, it is simple and economical, the supersaturation is relatively light, and the intermediate phase processing window is long [20–22]. For VQ, it can effectively achieve rapid nucleation to supersaturation, but with the increase in the film area, it has higher requirements for the vacuum system [19, 23, 24]. From the perspective of industrialization, GQ can be easily integrated in a production line and can be better combined with some in situ technologies to real-time monitoring, such as in situ X-ray diffractometer, in situ grazing incidence wide-angle X-ray scattering, and in situ photoluminescence. It is more in line with the demand of perovskite industrialization of cheap photovoltaic technology in principle [19, 20, 22, 25]. In addition, additive is a simple but effective strategy to optimize the intermediate phase through the formation of Lewis acid–base adduct or act as the sacrificial agent to assist crystallization [26–28]. It seems that the same additives can only achieve the best positive effect under specific perovskite components, solvent system, and preparation process conditions. That is interesting but very little research has been carried out to explore the reason. The above factors are related to the crystallization dynamics of the perovskite films; thus, it is natural to deduce that the role of additives is related to the film growth dynamics and the final quality of perovskite crystals.

In this work, the difference of additive distribution in the blade-coated perovskite thin film was studied based on GQ and VQ processes. As a result, the perovskite films incorporated by 1,3-bis(4-methoxyphenyl)thiourea (BM-TU) at buried interface exhibit excellent crystalline quality that contributing a certified PCE of 23.75% for the inverted PSC. More importantly, the champion PSC was also certified to have a constant PCE of 23.46% over 300 s at the maximum power point tracking (MPPT). Further more, we scaled up the blade-coated perovskite thin films to 10 × 10 cm^2^, and the perovskite solar module (PSM) yielded a PCE of 20.18% with an aperture area of 60.84 cm^2^, which is also among the highest reports to date.

## Experimental Section

### Materials

All chemicals in the experiment were used directly without further purification, including: [2-(3,6-dimethoxy-9H-carbazol-9-yl)ethyl]phosphonic acid (MeO-2PACz, > 98.0%, TCI), Al_2_O_3_ (30 nm, 20 wt% in isopropanol, Sigma-Aldrich), thiourea (TU, AR, 99%, Macklin), 1,3-bis(4-methoxyphenyl)thiourea (BM-TU, 98%, Aladdin), CsI (≥ 99.99%, Xi'an Yuri Solar Co., Ltd.), FAI (≥ 99.5%, Xi'an Yuri Solar Co., Ltd.), PEAI (≥ 99.5%, Xi'an Yuri Solar Co., Ltd.), PbI_2_ (≥ 99.99%, Xi'an e-Light New Material Co., Ltd.), PbCl_2_ (≥ 99.99%, Xi'an e-Light New Material Co., Ltd.), MACl (≥ 99.995%, Xi'an e-Light New Material Co., Ltd.), C_60_ (≥ 99%, Xi'an Yuri Solar Co., Ltd.), N,N-dimethylformamide (DMF, 99.8%, Sigma-Aldrich), 1-methyl-2-pyrrolidinone (NMP, 99.9%, Aladdin), isopropanol (IPA, ≥ 99.9%, Aladdin), and ethanol (EtOH, 99.5%, Aladdin).

### Density Functional Theory Calculations

Spin-polarized density functional theory (DFT) calculations were performed by employing the CP2K quantum chemistry software package. The Perdew–Burke–Ernzerhof (PBE) parametrization of generalized gradient approximation (GGA) was adopted to describe the exchange–correlation interactions in Hamiltonian. The GTH potential and Molopt basis set (DZVP-MOLOPT-SR-GTH) with a cutoff energy of 350 Ry was used to calculate. The Grimme’s D3 type of the semiempirical method was taken into account to depict the van der Waals (vdW) interactions. Both atomic position and cell parameters were relaxed until the max force is lower than 4.5E^−4^ Ha/bohr. Electrostatic potential was calculated by a self-consistent calculation. A three-layer slab with a p(3 × 3) supercell was used to simulate the alpha-FAPbI_3_ (100) surface. A five-layer slab with a p(6 × 6) supercell was used to simulate the Al_2_O_3_ (001) surface. We evaluated the adsorption interaction of TU, BM-TU–N, BM-TU–O, BM-TU–S on alpha-FAPbI_3_ (100), and BM-TU–O, BM-TU–S on Al_2_O_3_ (001) by the binding energy (Δ*E*_bind_). The binding energy was calculated as follows: Δ*E*_bind_ = *E*_(mole/surface)−_*E*_(surface) −_*E*_(mole)_, where *E*_(mole/surface)_, *E*_(surface)_, and *E*_(slab)_ are the DFT energies of the adsorbed molecule on surfaces, clean surfaces, and molecule, respectively.

### Device Fabrication

The PSCs were prepared with a structure of ITO/MeO-2PACz@Al_2_O_3_/(BM-TU or TU)/perovskite/C_60_/SnO_2_/Ag. It was worth noting that for substrates of different sizes, perovskite layers were prepared by blade coating. First of all, ITO glass substrates were cleaned ultrasonically with windshield washer fluid, deionized water, and ethanol (15 min each). After drying in the oven, they were further treated by UV-ozone for 15 min before use. Next, the SAM solution (0.3 mg mL^−1^ in ethanol) and Al_2_O_3_ solution (1 mL 20 wt% colloidal Al_2_O_3_ was diluted in 50 mL IPA) were deposited successively on the ITO glass substrates at 3500 rpm for 30 s and then annealed at 120 °C for 10 min, respectively. To prepare the interconnect layer BM-TU film, the BM-TU solution was deposited on the surface of the Al_2_O_3_ at 3500 rpm for 30 s and annealed at 120 °C for 10 min. For the perovskite absorption layers, the Cs_0.08_FA_0.92_PbI_3_ precursor solution was prepared by dissolving CsI (24.9 mg), FAI (89.7 mg), PbCl_2_ (25.2 mg), PbI_2_ (553 mg), and MACl (5.6 mg) into mixed solvents of DMF and NMP with volume ratio of 6:1, and then heated at 55 °C till completely dissolved. For the perovskite thin film with small-size substrate (2.5 × 2.5 cm^2^) or large-size substrate (10 × 10 cm^2^), the perovskite absorber layer was subsequently deposited using the air extraction-assisted blade-coating method. The PEAI solution (2 mg mL^−1^ in IPA) was spin coated onto the perovskite film surface at 4000 rpm for 30 s, followed by annealing at 120 °C for 10 min. The C_60_ film was evaporated to a thickness of 40 nm. The SnO_2_ film was deposited by atomic layer deposition with Tin (IV) dimethylamide as Sn source and H_2_O as O source at 110 °C for 200 cycles. All the devices for performance and stability evaluation were tested without encapsulation. Finally, an Ag electrode (100 nm) was evaporated by thermal evaporation under vacuum.

### Perovskite Film Fabrication

#### ***Solar Cells (2.5*** × ***2.5 cm***^***2***^***) Fabrication***

The deposition of the perovskite precursor films prepared in the paper was performed on a commercial blade coater (ZAA2300.H from ZEHNTNER) using a ZUA 2000.100 blade (from ZEHNTNER) at room temperature. Blade deposition was carried out using a 30 µL perovskite precursor solution on each 2.5 × 2.5 cm^2^ substrate. The coating speed was fixed at 3 mm s^−1^, and the gap for solution load between the substrate and blade was fixed at 250 µm. After the blade-coating process, the freshly coated liquid precursor film remained for 30 s, allowing the solution to evaporate slightly. Immediately afterward, the precursor film was blown dry with an air gun (~ 0.2 MPa) to obtain the intermediate phase of the perovskite film. For the vacuum quenching process, as soon as the blade coating was completed, the precursor film was transferred to a vacuum chamber, which was pumped to 1000 Pa within 15 s, and held at that pressure for 100 s. Subsequently, the films were transferred to a hot table and annealed at 120 °C for 10 min. The active area of the small-size perovskite solar cell was 0.09 cm^2^.

#### ***Mini-Modules (10*** × ***10 cm***^***2***^***) Fabrication***

The coating equipment and preparation process of large-size perovskite film was the same as that of small-area size. The difference was that 200 µL precursor solution was used and air drying with an air knife for blade deposition on each 10 × 10 cm^2^ substrate. Meanwhile, the gas pressure of the air knife was set at ~ 0.3 MPa, and the included angle between the substrate and air knife was about 60°. The aperture area of the large-size perovskite mini-module was 60.84 cm^2^.

### Laser Scribing Procedure

The ambient temperature of the laser processing was controlled to 24 ± 2 °C, and the humidity was 45 ± 5% RH. The P1 patterns, which used the Helios fiber laser processing system with a wavelength of 1064 nm and minimum pulse duration of 30 ns, were etched onto ITO glass substrates. Among them, the power and speed of the laser were 12.5 W and 1200 mm s^−1^, respectively. Using the Helios VIS laser processing system, the P2 and P3 patterns were etched at a wavelength of 532 nm and minimum pulse duration of 0.4 ns. The etching of P2 pattern occurred after the deposition of the ALD-SnO_2_ films, with a power of 0.25 W and a scanning speed of 680 mm s^−1^. At a power of 0.25 W and a scanning speed of 800 mm s^−1^, the P3 pattern was etched after the deposition of silver electrodes.

### Characterization

The current density–voltage (*J-V*) curves were characterized under ambient conditions at room temperature by using a digital source meter (Keithley 2400) and a Newport solar simulator (ORIEL-SOI3A) with AM 1.5 G spectrums. The light intensity on the sample was adjusted to AM1.5G one sun (100 mW cm^−2^) using a standard Si cell (91150 V). The external quantum efficiency (EQE) spectra of PSCs were measured in DC mode on a spectrum corresponding system (Enlitech QE-R), calibrated by Si reference solar cell. The morphology characterization of thin films and PSCs was measured by scanning electron microscope (SEM) (FEI Apreo LoVac). Carrier lifetime fluorescence spectra were measured by fluorescence lifetime imaging microscopy (FLIM) (PicoQuant, Micro Time200). The depth analysis of element distribution in perovskite device was obtained by the time-of-flight secondary ion mass spectrometry (TOF–SIMS) (ION TOF–SIMS 5). The atomic force microscope (AFM) images were obtained using NT-MDT NDTGRE. The X-ray photoelectron spectroscopy (XPS) characterizations were performed by Thermo Fisher Scientific K-ALPHA^+^, using the HeI (21.22 eV) emission line and Al Kα radiation (energy 1486.6 eV). The crystal structure was measured by Bruker D8 Advance X-ray diffractometer (XRD) with Cu Kα radiation at 40 kV and 40 mA. The steady-state photoluminescence (PL) and time-resolved photoluminescence (TRPL) spectra were obtained via an Edinburgh Instruments FLS1000 fluorescence spectrometer with a 450 nm picosecond pulsed laser. The PL mapping spectrum was acquired by a micro-confocal Raman spectrometer (WITec Alpha 300R) with an excitation wavelength of 532 nm. The optical properties of perovskite films were measured with a UV–Vis Cary 5000 spectrophotometer (Agilent technologies). The capacitance–voltage (C–V) measurements and voltage decay measurements were collected from electrochemical workstation (ZAHNER GIMPS, Germany).

## Results and Discussion

### Perovskite Thin Films Prepared by Different Extraction Methods

**Fig. 1:**
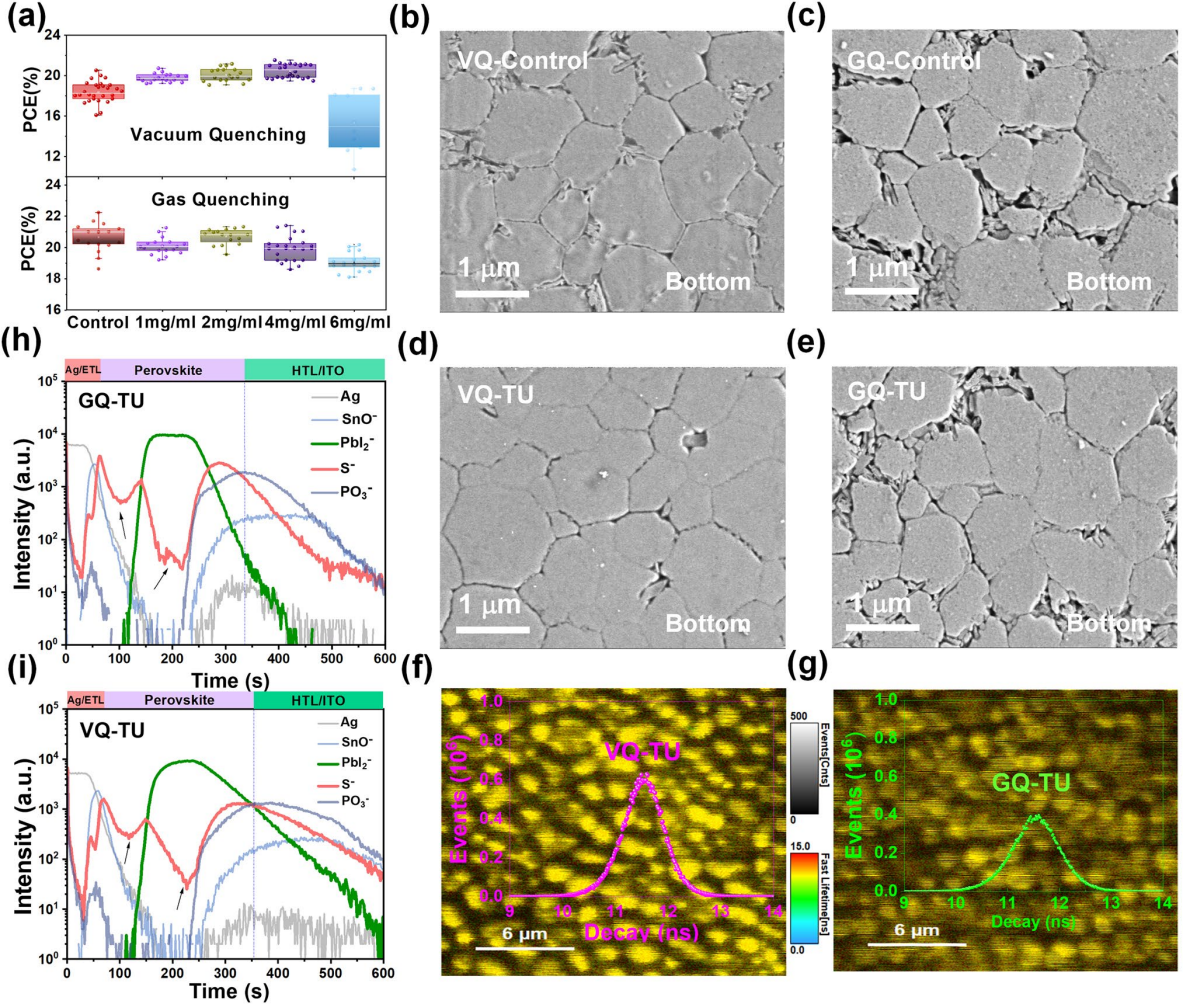
**a** PCE parameter statistics of PSCs prepared by VQ and GQ methods under different TU addition concentrations. Top-view SEM of buried interfacial perovskite film prepared by **b** VQ-w/o TU, **c** GQ-w/o TU, **d** VQ-TU, and **e** GQ-TU. Fluorescence lifetime imaging of the corresponding buried interfacial perovskite film prepared by **f** VQ-TU and **g** GQ-TU. The TOF–SIMS data of deposited devices during different drying processes **h** GQ-TU and **i** VQ-TU

The initial reason for our study of the difference between the preparation of perovskite films by GQ and VQ processes was the discovery that the same additive was added to the perovskite precursor solution, but the properties of the PSCs prepared by the two methods were different. As shown in Fig. 1a, adding a certain concentrations of thiourea (TU) is able to improve the device efficiency by using the VQ method, but the effect is not obvious by using the GQ method. This is mainly manifested in the failure on the filling factor (*FF*) and open-circuit voltage (*V*_OC_) in the GQ process (Fig. S1). For the short-circuit current (*J*_SC_), the addition of TU (4 mg mL^−1^) in different extraction methods show a similar increase with the enhancement of EQE range at wavelength of 350–500 nm (Fig. S2). Regardless of the effects of parasitic absorption of front interface layer, it is most likely that the pre-contact interface has changed, which made us curious about the state of the perovskite film buried at the bottom interface. SEM technology was used to observe the buried interface morphology. Large amount of voids at the grain boundaries are found at the buried interface of films (Figs. 1b, c and S3), and the TU additive is favorable for decrease them (Figs. 1d, e and S4). The difference is that the film prepared by GQ method has more voids, and they are not easy to be eliminated despite the addition of TU. It has been reported that the pre-crystallization of perovskite on top of wet film slows down the solvent evaporation at the buried interface, making it difficult for the trapped solvent at the interface to effectively escape and eventually form a void [29, 30]. The fluorescence lifetime imaging microscopy (FLIM) shows that the presence of void severely affects the PL lifetime throughout the film due to the trap-assisted recombination (Fig. 1f, g) [31]. In addition, the morphology of the top surface of perovskite films is also affected by different extraction methods (Figs. S5 and S6). The perovskite grains obtained by GQ method are larger than those obtained by VQ method (Fig. S5), which is verified by both with/without TU-modified perovskite films. This result may be caused by the following factors: i) the supersaturation rate of common quenching methods at critical concentration, GQ (~ 10^−5^ − 10^0^ s^−1^) is significantly faster than VQ (~ 10^−3^ − 10^−1^ s^−1^), leading to fewer number of nuclei, tending to strong Oswald ripening [19]. ii) The GQ process may result in incomplete extraction of solvent near the buried interface that promotes the second growth of perovskite grains [32, 33].

The above results and analysis suggest that VQ and GQ may result in the intermediate phase films with different crystal nucleus distributions. Thus, we performed the time-of-flight secondary ion mass spectrometry (TOF–SIMS) to characterize the distribution of TU in perovskite films so as to infer the crystallization dynamics evolution process. The strong coordination between TU and PbI_2_ can delay the crystallization of perovskite, and the TU will be excluded from the perovskite lattice through intramolecular exchange. Therefore, we can judge the order of crystallization by the level of TU content detected in the film. The initial crystallized grains would have less TU than the rest uncrystallized wet film during the preparation process of perovskite films. As shown in Fig. 1h, i, the TU intensity has two troughs, which are distributed near the longitudinal top plane and the intermediate bulk, respectively, indicating that the grains grow in layers and eventually consolidated into large grains by van der Waals ripening. We use PO_3_^−^ from the ultra-thin self-assembled monolayers MeO-2PACz to determine the location of the buried interface. It is interesting that the intensity peak location of TU in GQ films is farther than that in VQ films from the buried interface, which suggests that the film prepared by GQ is easier to crystallize at the buried interface during the annealing process on the hotplate. It is the reason why there are still more holes in the buried interface of perovskite films prepared by GQ, because the lack of TU-PbI_2_ complex not only increases the volatile solvent content but also cannot delay crystallization to form compact thin film.

### Crystallization Dynamics of Different Extraction Methods

**Fig. 2:**
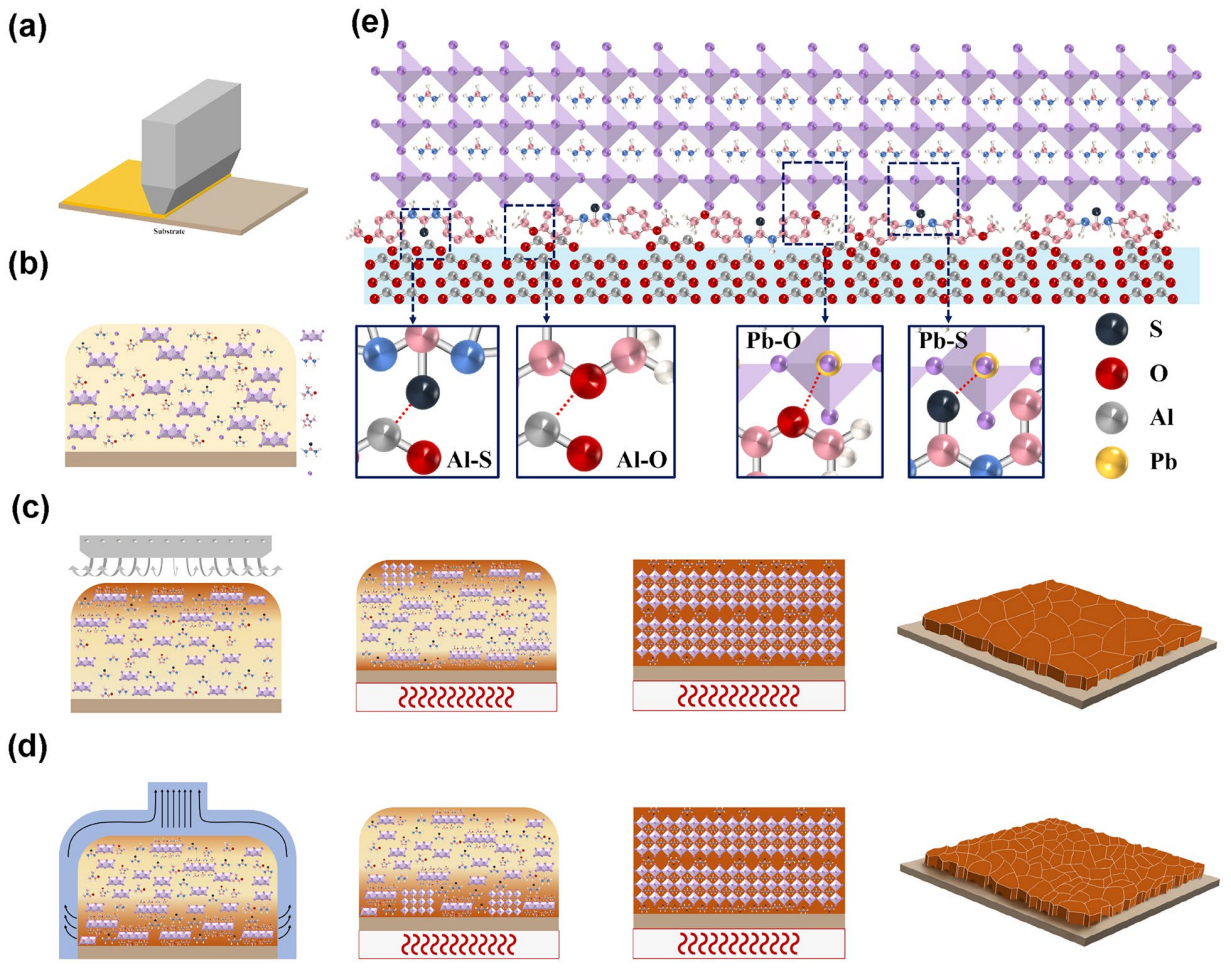
**a** Preparation of perovskite film by blade-coating method. **b** Schematic representation of perovskite precursor fluid membrane covering. Schematic diagram of the perovskite nucleation/growth model under **c** GQ method and **d** VQ method. **e** Schematic diagram of intrfacial defect passivation by BM-TU

Here, we proposed a schematic diagram of the growth dynamic difference of VQ- and GQ-prepared perovskite films. Figure 2a, b shows the model diagram of perovskite film prepared by blade-coating method and the schematic diagram of molecular distribution of perovskite liquid film, respectively. For the GQ process (Fig. 2c), the air flow will first act on the top surface of the perovskite wet film, so that the wet film will be preferred to form an intermediate phase on the top surface [32]. Further, with the increase in GQ time, the nucleation of intermediate will occur from top to bottom with the infiltration of air flow. However, due to the deepening of the curing of the intermediate phase on the top surface, the further infiltration of the air into the buried interface will be hindered [32]. It may lead to a little amount of pre-crystallized nucleus present at the bottom interface with incomplete extraction of solvent. During the stage of the annealing process, the bottom of the perovskite film is preferred to be heated first, as a result the intermediate phase of the top and bottom surfaces crystallized at the same time, but the poor volatilized underlying solvent leads to the formation of holes. For the VQ method (Fig. 2d), the solvent extraction leads to an overall increase in solution concentration in the wet film. The top surface of the wet film is preferentially extracted to form the intermediate phase, and the solvent that continues to be extracted from the bottom will re-dissolve part of the intermediate phase on the top surface with the increase in further vacuum extraction time. It may lead to the intermediate phase crystalline grains at top surface which are not dense that facilitates the volatilization of solvent near the buried interface during the annealing process and formation of less voids. When the TU additive is added, the TU content is squeezed to the top surface, the junction of layered grain, and the buried interface. It can be deduced that inhibiting the nucleation of the buried bottom interface but reducing the solvent residue at the same time during the solvent extraction stage is beneficial to preparing excellent buried bottom interface with less holes. Reducing the adduct between solvent and PbI_2_ while using the solid TU to delay the crystallization of perovskite at buried interface is assumed to be a promising candidate strategy.

However, the TOF–SIMS results in Fig. 1h have shown that the TU accumulates at a certain distance from the burial interface in the GQ method. It requires a pre-deposited TU layer. Thus, we designed a modified TU molecule with anchoring groups, which not only enhances the role of TU, because the electron conjugation of π on the benzene ring can enhance the electron donating ability of TU, but also enables the methoxy group to self-assemble with Al_2_O_3_ nanoparticles, so that TU will not dissolve when the perovskite solution is deposited [34–36]. In summary, the constructed multi-site passivation groups can be assembled on the Al_2_O_3_ layer and act on the perovskite layer to form the interconnect layer as shown in Fig. 2e.

### Molecular Design and Interconnect Layer Construction

**Fig. 3:**
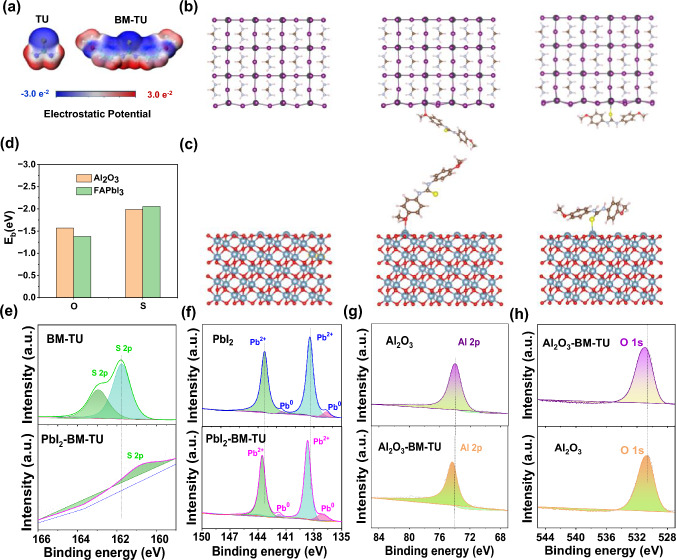
**a** Electrostatic potential of TU and BM-TU. The optimized structures of defective for **b** FAPbI_3_, FAPbI_3_–BM-TU(_O–Pb_^2+^), and FAPbI_3_–BM-TU(_S–Pb_^2+^). **c** Al_2_O_3_, Al_2_O_3_–BM-TU(_O–A_^3+^), and Al_2_O_3_–BM-TU (_S–Al_^3+^). **d** Calculated data of the binding energies. XPS spectra of **e–f** perovskite–BM-TU and **g-h** Al_2_O_3_–BM-TU

The electrostatic potential (ESP) of TU and BM-TU was calculated by DFT, as shown in Fig. 3a. The main difference between the two molecules is that the introduction of methoxy group and benzene ring increases the electron donor site of the molecule, which makes that BM-TU has the characteristics of multi-site and strong passivation. To shed deep insight into the BM-TU–perovskite and BM-TU–Al_2_O_3_ interaction, the DFT calculations were applied to evaluate the surface adsorption of individual passivated atoms. The corresponding optimized structure of the simulation is shown in Figs. 3b, c and S7, and the corresponding binding energy (*E*_b_) was calculated and summarized in Table S1. Among these functional groups of the BM-TU molecule, the -NH group is not adsorbed to the Al_2_O_3_ surface, but can be attached to Pb^2+^ by the formation of the hydrogen-bonding interactions [36]. Moreover, the values of *E*_b(S–Al_^3+^_)_ are significantly lower than that those of *E*_b(S–Pb_^2+^_)_, as shown in Fig. 3d, indicating that the -S group prefers to bind with perovskite layer. As for the -OCH_3_ group, the calculated results show that it is more inclined to bind with Al_2_O_3_ (*E*_b(O–Al_^3+^_)_ > *E*_b(O–Pb_^2+^_)_). Similarly, the XPS also confirmed that C = S and -OCH_3_ group in BM-TU molecules can bind to perovskite and Al_2_O_3_ layers, respectively (Figs. 3e–h and S8). And the specific data are discussed in detail in Supplementary Note S1. The change of C = S tensile vibration of BM-TU film also confirms that partial electron cloud migration from C = S to S⋅Pb (Fig. S9). In addition, the MeO- has excellent adsorption as a self-assembled group onto metal oxide [37]. In addition to theoretical and literature support, we observed that adding BM-TU to Al_2_O_3_ nanoparticle solution leads to the precipitation of nanoparticle excludes the influence of the solvent, thus revealing the interaction between them (Fig. S10). In short, the BM-TU molecule may form a bridging layer between the Al_2_O_3_ and perovskite films, which would facilitate to keep the passivator at the buried interface.

### Photovoltaic Performance of PSCs with Tailor Designed Additive

**Fig. 4:**
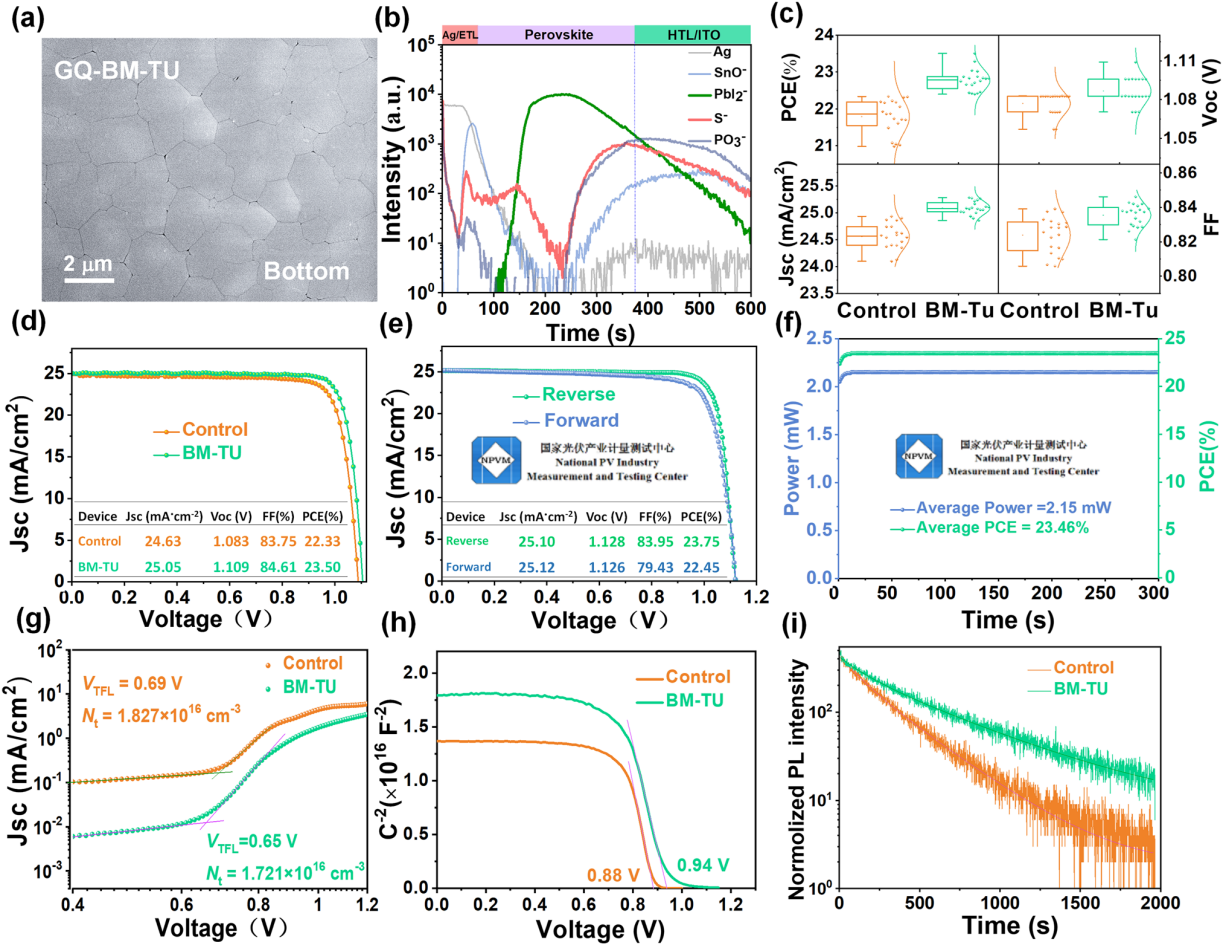
**a** Buried SEM images of perovskite films with BM-TU treatments. **b** TOF–SIMS data of deposited devices with BM-TU treatments. The control and BM-TU-modiffed device for **c** statistical distribution of the PCE, *V*_OC_, *J*_SC_, and *FF*. **d** Reverse scanning *J–V* curve. The certified results recorded by the Chinese national PV industry measurement and testing center (NPVM), under AM1.5G 100 mW cm^−2^ irradiation: **e** *I–V* curve from reverse and forward scan, **f** steady-state power output, **g** dark *I–V* curves of the hole-only, **h** Mott–Schottky plots where the solid lines were fitted linearly, and **i** TRPL spectra

As expected, the TU signal is strongest at the buried interface from the TOF–SIMS results, and the perovskite thin film gains superior morphology with compact coverage and pinhole-free buried interface from the SEM results (Figs. 4a, b and S11). The schematic diagram and position distribution of BM-TU in the nucleation and crystallization process of perovskite films are shown in Fig. S12. The BM-TU molecule at the buried bottom interface is anchored on Al_2_O_3_, which inhibiting the nucleation of the buried bottom interface but reducing the solvent residue at the same time during the solvent extraction stage is beneficial to preparing excellent buried bottom interface with less holes. Meanwhile, the BM-TU is not fully anchored to the substrate, with some of the BM-TU dissolves into the perovskite ink, participating in the crystallization and growth of the film. As the perovskite crystallizes and grows, BM-TU is extruded to the top surface of the perovskite film. The X-ray diffraction (XRD) patterns show that the (110) and (220)-preferential orientation peak intensity is significantly enhanced after the incorporation of BM-TU (Fig. S13). The absorption data of perovskite films also support this view (Fig. S14). The ultraviolet photoelectron spectroscopy (UPS) was carried out to determine the energy level change of the interface (Fig. S15). The valence band position of the BM-TU-modified perovskite film is -5.908 eV, which is almost consistent with the film without BM-TU (-5.895 eV). Additionally, the PL mapping images and top-view SEM images also indicate that both uniformity and optoelectronic quality of perovskite thin film that grown on BM-TU are notably improved [38] (Figs. S16 and S17). And the specific datas are discussed in detail in Supplementary Note S2. To evaluate the impact of introducing BM-TU at the buried interface on the device photovoltaic performance, p-i-n PSCs with a device structure of ITO/MeO-2PACz@Al_2_O_3_/(BM-TU or TU)/perovskite/C_60_/SnO_2_/Ag were fabricated. The corresponding solar cell parameters of 20 devices were counted, as shown in Figs. 4c, S18, and S19. TU molecules that without MeO- anchoring groups will be dissolved more into perovskite inks near the buried interface, forming a bulk doping instead of aggregating at the buried interface. Attributed to MeO- anchoring groups in BM-TU, it is impossible to create a strong bridge with Al_2_O_3_ and perovskite at the same time and gain the best passivation effect of the buried interface. Here, the PSCs with BM-TU demonstrate a champion PCE of 23.51% with a *J*_SC_ of 25.05 mA cm^−2^, a *V*_OC_ of 1.109 V, and an *FF* of 0.846, which is higher than the control device with PCE of 22.33% (Fig. 4d). The external quantum efficiency (EQE) spectra of the corresponding PSCs with and without BM-TU modification show that the optimized device has a significant boost over the entire absorption band compared to the control device with the integrated *J*_SC_ values are 23.75 and 24.15 mA cm^−2^, respectively (Fig. S20). We also sent our champion PSC (Fig. S21) to the Chinese national PV industry measurement and testing center (NPVM) for independent certification. The certified PCE under reverse scan (*V*_OC_ to *J*_SC_) is 23.75% (Figs. 4e and S22), the aperture area of the PSC is 0.0916 cm^2^, and a *J*_SC_ of 25.10 mA cm^−2^, *V*_OC_ of 1.128 V, and *FF* of 83.95% is observed. More importantly, the champion PSC was also certified to have a constant PCE of 23.46% over 300 s at the maximum power point tracking (MPPT) (Figs. 4f and S23). To quantify the trap density in perovskite films, the space-charge limited current (SCLC) measurement of the HTL-only devices (ITO/MeO-2PACz@Al_2_O_3_/(BM-TU)/perovskite/PTAA/Ag) was performed. From the dark *J–V* characteristics obtained in Fig. 4g, the *V*_TFL_ of pristine device and with BM-TU modification device are 0.69 and 0.65 V, respectively. The calculated *N*_trap_ are 1.827 × 10^16^ and 1.721 × 10^16^ cm^−3^ for devices without and with BM-TU modification, respectively, indicating a substantial reduction of trap states [39]. As is shown in Fig. 4h, from the Mott–Schottky plots, the *V*_bi_ of with BM-TU-modified device (0.94 V) is higher than that of control-based (0.88 V) device, which increased the driving force of photogenerated carrier dissociation and facilitated the formation of an extended depletion region to effectively inhibit recombination [40]. To further investigate the charge transfer and recombination kinetics of perovskite films, we performed the steady-state PL and TRPL using a structure of ITO/Al_2_O_3_/perovskite. The perovskite on BM-TU-modified Al_2_O_3_ shows higher PL intensity compared to that on pristine Al_2_O_3_ at 804 nm (Fig. S24), indicative of the non-radiative recombination within the bare perovskite film was significantly suppressed [41]. In addition, the average lifetime (${\tau }_{ave}$) of without modified perovskite film has risen from 297.68 to 530.99 ns after BM-TU modification (Fig. 4i and Table S2), and the longer carrier lifetime proves the excellent passivation ability of BM-TU modification [41, 42]. Figure S25 reveals the plot of the *V*_OC_ versus the common logarithm of light intensity, where the slope (*S*) of the two curves can be obtained by performing a linear fit [39]. After BM-TU passivation, the *n* value decreased significantly from 1.43 to 1.30, indicating that the recombination loss decreased significantly in the device.

### Photovoltaic Performance and Operational Stability of PSMs

**Fig. 5:**
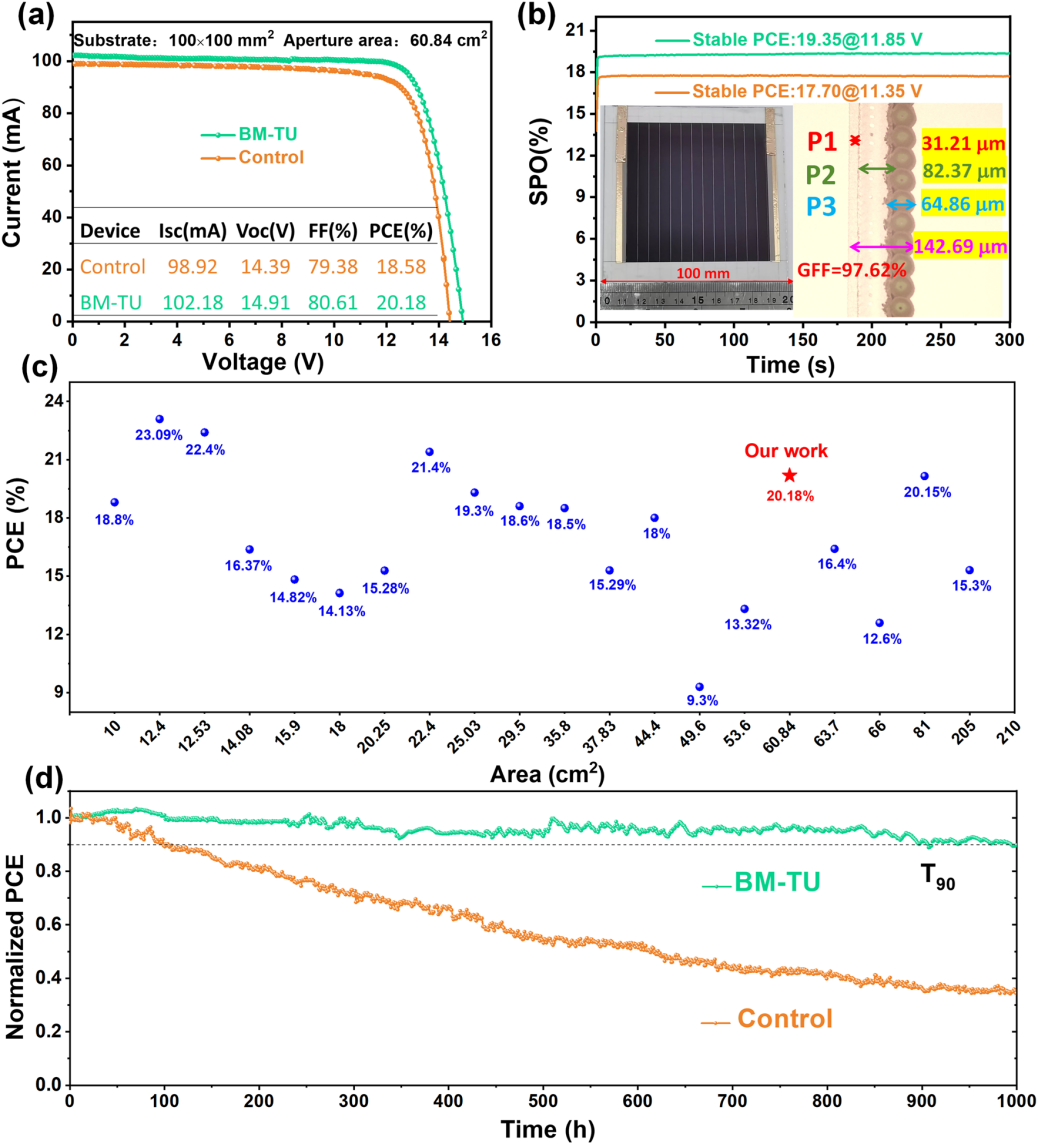
**a** *J–V* characteristics of the champion perovskite solar module with an aperture area of 60.84 cm^2^. **b** Steady-state PCE of the champion perovskite solar module (Inset 1 is photograph of the fabricated perovskite solar module; inset 2 is microscope images of the P1, P2, and P3 scribe lines in the dead area of PSMs). **c** Summary of PCE for solar modules based on blade coating in recent years. **d** MPPT performance of the perovskite solar module

The improvement of film crystallization at the buried interface by BM-TU will be more conducive to the scalable preparation of modules for practical commercial applications. We prepared 10 × 10 cm^2^ perovskite solar modules (PSMs) consisting of 13 subcells of 6 mm width in series with the design of the P1–P2–P3 pattern are shown in Fig. S26. Encouragingly, the champion PSMs yield a PCE of 20.18% with a *V*_OC_ of 14.91 V, a *I*_SC_ of 102.18 mA, and a *FF* of 80.61% (Fig. 5a) superior to the control ones (PCE = 18.58%, *V*_OC_ = 14.39 V, *I*_SC_ = 98.92 mA, and *FF* = 79.38%). Figure S27 shows the forward and reverse scan *J−V* test results of perovskite solar modules and calculated the hysteresis index (HI). The BM-TU-modified module shows a HI of 3.2%, which is significantly lower than the results of control-based devices (6.1%). As shown in Fig. 5b, PSMs present a smooth and continuous output PCE of 19.35% (with BM-TU) and 17.70% (without BM-TU) within 300 s. The insets exhibit the photo and the laser scribing microscope photo. The width of dead area is 142.69 μm, and the geometric filling factor (GFF) of the PSM is calculated to be 97.62%. We summarize the representative works that reported in recent years for large-area modules based on blade coating (Fig. 5c and Table S3). Encouragingly, our work is among the highest PCEs for the large-area device with area exceeding 50 cm^2^. The PSMs with BM-TU possess remarkable continuous operation stability for MPPT of T_90_ > 1000 h in ambient air (Fig. 5d). For comparison, the PSM without BM-TU degrades to < 40% of the initial PCE after 1000 h operation. This result provides a potential and opportunity to satisfy the IEC61215 demands. The BM-TU-modified PSCs also show better thermal stability (at 85 °C without encapsulation) as it can retain 91.0% of the initial PCE after aging for 14 days, whereas the control device only retained 71.5% (Fig. S28). The optimized unencapsulated device also showed excellent storage stability, maintaining an initial efficiency of more than 94% for a year (Fig. S29).

## Conclusions

In this work, inspired by the results showing differences in the photovoltaic performance of PSCs prepared by VQ- and GQ- with the same additive, we carried out a study to infer the crystallization dynamics process. We found that these two pre-crystallization processes can lead to different crystallization dynamics so as to the differences of additive distribution. In order to solve the problem of inferior buried interface in the GQ-prepared perovskite thin film, we tailor-designed thiourea variant molecular material (BM-TU) with anchoring groups, which not only enhances the role of TU, but also enables the self-assemble with underlying layer. The champion PSCs demonstrate a certified efficiency of 23.75%, and the PSM yields an efficiency of 20.18% with an aperture area of 60.84 cm^2^, which are among the highest reports to date. The PSM possesses remarkable continuous operation stability for MPPT of T_90_ > 1000 h in ambient air.

## Supplementary Information

Below is the link to the electronic supplementary material.

Supplementary file1 (DOCX 20502 kb)
