# Supplementary material for: Efficient and Stable Perovskite Solar Cells and Modules Enabled by Tailoring Additive Distribution According to the Film Growth Dynamics
Source: Nanomicro Lett. 2024 Oct 15;17:39. doi: 10.1007/s40820-024-01538-7 (PMC11480303; doi:10.1007/s40820-024-01538-7)
Supplement: Supplementary file 1 — Supplementary file1 (DOCX 20502 kb) [file 40820_2024_1538_MOESM1_ESM.docx]

Supporting Information for

**Efficient and Stable Perovskite Solar Cells and** **Modules Enabled by** **Tailoring Additive Distribution According to the Film Growth Dynamics**

Mengen Ma^1^*^,^*^2,#^, Cuiling Zhang^3,#^, Yujiao Ma^1^*^,^*^2^, Weile Li^1^*^,^*^2^, Yao Wang^1^*^,^*^2^, Shaohang Wu^1^*^,^*^2^*^,^*^3^, Chong Liu^1^*^,^*^2^*^,^*^3,^*, Yaohua Mai^1^*^,^*^2^*^,^*^3^

^1^Institute of New Energy Technology, College of Physics & Optoelectronic Engineering, Jinan University, Guangzhou 510632, People’s Republic of China

^2^Key Laboratory of New Semiconductors and Devices of Guangdong Higher Education Institutes, Jinan University, Guangzhou 510632, People’s Republic of China

^3^Guangdong Mellow Energy Co., Limited, Yuanming Road, Zhuhai 519075, Guangdong, People’s Republic of China

^#^Mengen Ma and Cuiling Zhang contributed equally to this work.

*Corresponding author. E-mail: [chongliu@jnu.edu.cn](mailto:chongliu@jnu.edu.cn) (Chong Liu)

# S1 Supplementary Figures and Tables

**Fig. S1** The parameter statistics of PSCs prepared by VQ and GQ method under different TU addition concentrations for **a** *J*_SC_, **b** *V*_OC_, **c** *FF*

**Fig. S2** EQE and integrated current density spectra of the PSCs based on different TU addition concentration **a** VQ method, **b** GQ method

**Fig. S3** Cross-sectional SEM images of PSCs based on control prepared by GQ and VQ methods

**Fig. S4** Cross-sectional SEM images of PSCs with TU prepared by GQ and VQ methods

**Fig. S5** Top-surface SEM images and the corresponding crystal size statistics of perovskite film prepared by **a** GQ-w/o TU, **b** GQ-TU, **c** VQ-w/o TU, **d** VQ-TU

**Fig. S6** Top-surface AFM images of the perovskite films with TU prepared by **a** GQ method, **b** VQ method

**Fig. S7** The optimized structures of defective for FAPbI_3_-TU (_S-Pb_^2+^), FAPbI_3_-BM-TU(_N-Pb_^2+^)

**Fig. S8** The XPS spectra of **a** O 1s, **b** N 1s, **c** I 3d in perovskite films before and after BM-TU treatment

**Supplementary Note S1**

The change of element valence is probed through X-ray photoelectron spectroscopy (XPS) characterization. As shown in Fig. 3e, f, the pure BM-TU shows two S 2*p* peaks at 162.4 and 161.3 eV, while the BM-TU-PbI_2_ only has a strongly inhibited S 2*p* peak at 160.6 eV, indicating that BM-TU strongly interacts with PbI_2_. At the same time, the BM-TU addition leads to pronounced shifts of Pb 4*f* peaks from 142.6 and 137.8 eV to 143.0 and 138.2 eV, respectively. The shifts towards lower binding energies suggest an increased electron cloud density at Pb^2+^, which is stemmed from the acceptance of electrons from S and thus forming strong Pb-S bonds [S1]. Similarly, after BM-TU modification, the O 1*s* peak was split and transferred to a lower binding energy, and the N 1*s* peak in BM-TU was inhibited, which further confirmed that the customized multi-site functional group reacted with PbI2 (Fig. S8a, b) [S2]. Meanwhile, I 3*d* peak displays a tiny shift, indicating that the chemical environment of I is also varied (Fig. S8c). It may be due to the change of Pb^2+^ coordination environment that affects the Pb-I bond coupling, and also from the interaction between BM-TU and I.^[1]^ The same evidence has been shown for the binding of BM-TU molecules to the surface of Al_2_O_3_ films. As revealed in Fig. 3g, h, the O 1*s* peak on BM-TU moves in the direction of lower binding energy, while the Al 2*p* peak in Al_2_O_3_ moves in the direction of higher binding energy, indicating the interaction between BM-TU and Al_2_O_3_ film.

**Fig. S9** The FTIR spectra of C=S stretching vibrations for BM-TU and BM-TU@Perovskite

**Fig. S10** The optical photo of solution for Al_2_O_3_/IPA, Al_2_O_3_/IPA+DMF, BM-TU- Al_2_O_3_/IPA+DMF

**Fig. S11** The cross-sectional SEM image of PSC with BM-TU


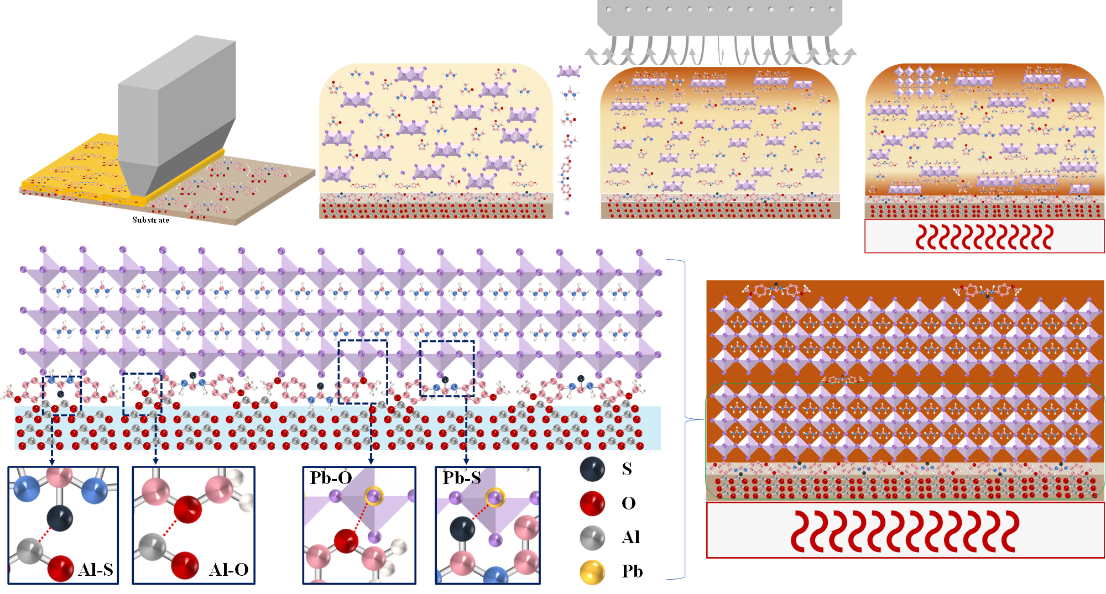


**Fig. S12** The flow diagram of perovskite film prepared by gas extraction under BM-TU modification

**Fig. S13** The XRD patterns of the top and bottom film surfaces with and without BM-TU modification

**Fig. S14** The absorption data of the top perovskite films without and with BM-TU modification

**Fig. S15** The UPS spectra of perovskite films with or without TU modification

**Fig. S16** Confocal PL mapping (5 × 5 μm^2^) of **a** control and **b** BM-TU-modiffed perovskite flms at the buried bottom interface

**Fig. S17** SEM crystal size labeling diagrams **a** control, **d** BM-TU-modified, Crystal size statistics **b** control, **e** BM-TU-modified, AFM maps of **c** control, **f** BM-TU-modified perovskite film on top surface

**Supplementary Note S2**

Figure S16 shows the 2D, 3D confocal photoluminescence mapping and CCD distribution of the bottom perovskite films with and without BM-TU modification respectively. The PL mapping of BM-TU modified perovskite film has relatively uniform and strong spatial PL signal, indicating reduced nonradiative recombination after BM-TU modification [S3, S4]. The SEM images of Fig. S17 show that the perovskite films with or without BM-TU modification all present pinhole-free crystals with high crystallinity, smooth surface and large grains. From the crystal distribution diagram obtained by SEM images, it can be seen that the grain size of the perovskite film deposited without BM-TU modification is randomly distributed, with an average of 954 nm (Fig. S17a, b). Surprisingly, in the BM-TU-modified perovskite film, the grain size becomes more uniform and the average size is greatly enhanced to 1257 nm (Fig. S17 d, e). The surface roughness of perovskite film was revealed by atomic force microscope (AFM), which shows a significantly increase from 30.607 nm to 35.408 nm after BM-TU modification (Fig. S17c, f), providing better interface contact for efficient carrier transport and extraction processes in the device.

**Fig. S18** The individual parameters of photovoltaic performance of **a** control and **b** BM-TU-modiffed PSCs

**Fig. S19** The control, TU-modiffed and BM-TU-modiffed device for **a** statistical distribution of the PCE, *V*_OC_, *J*_SC_ and *FF*. **b** Reverse scanning *J−V* curve

**Fig. S20** The control and BM-TU-modiffed device for EQE and integrated current density spectra

**Fig. S21** The reverse and obverse side of the measured sample


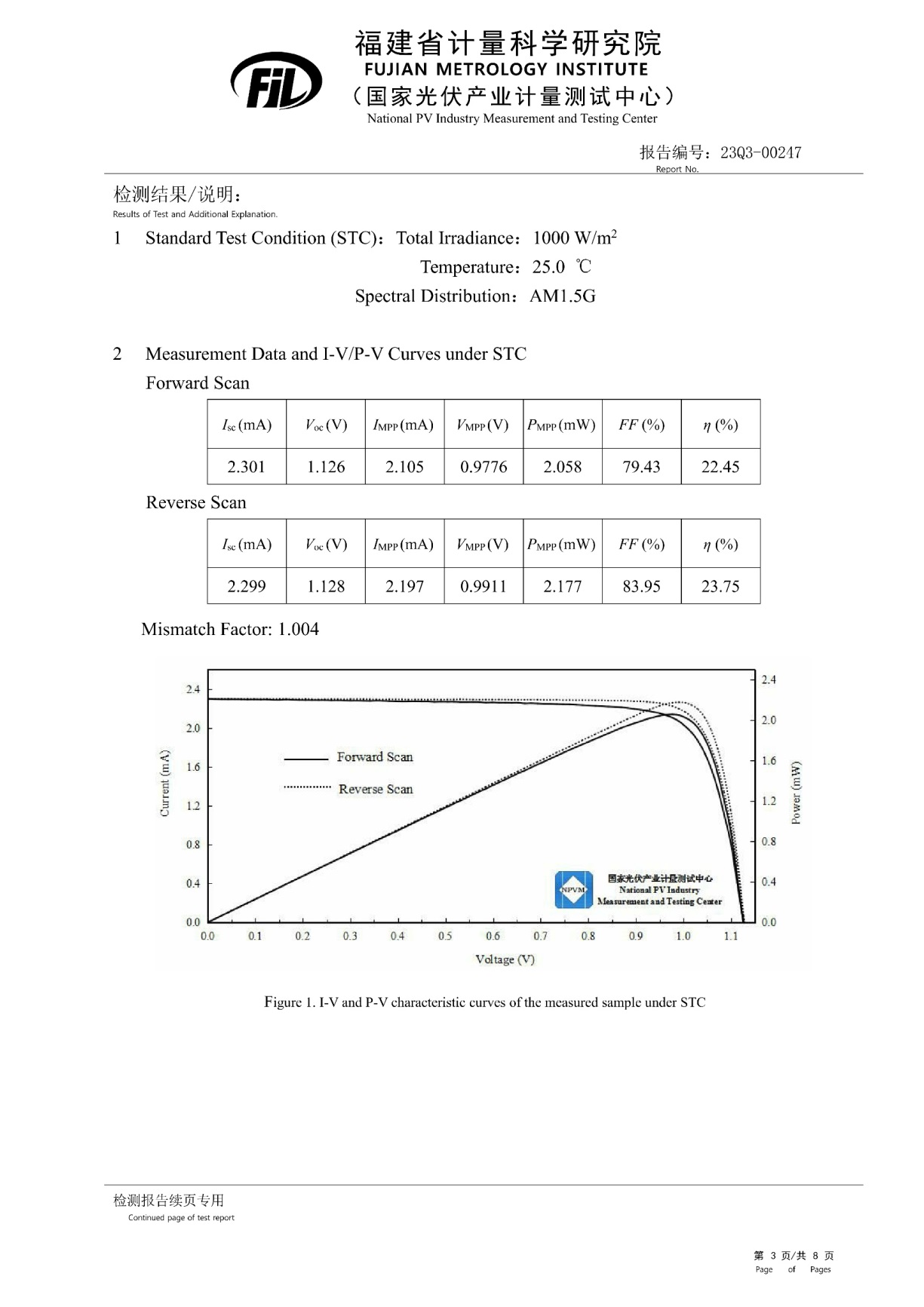


**Fig. S22** The certification report with the *I-V* curves of our best PSC determined by the Chinese national PV industry measurement and testing center


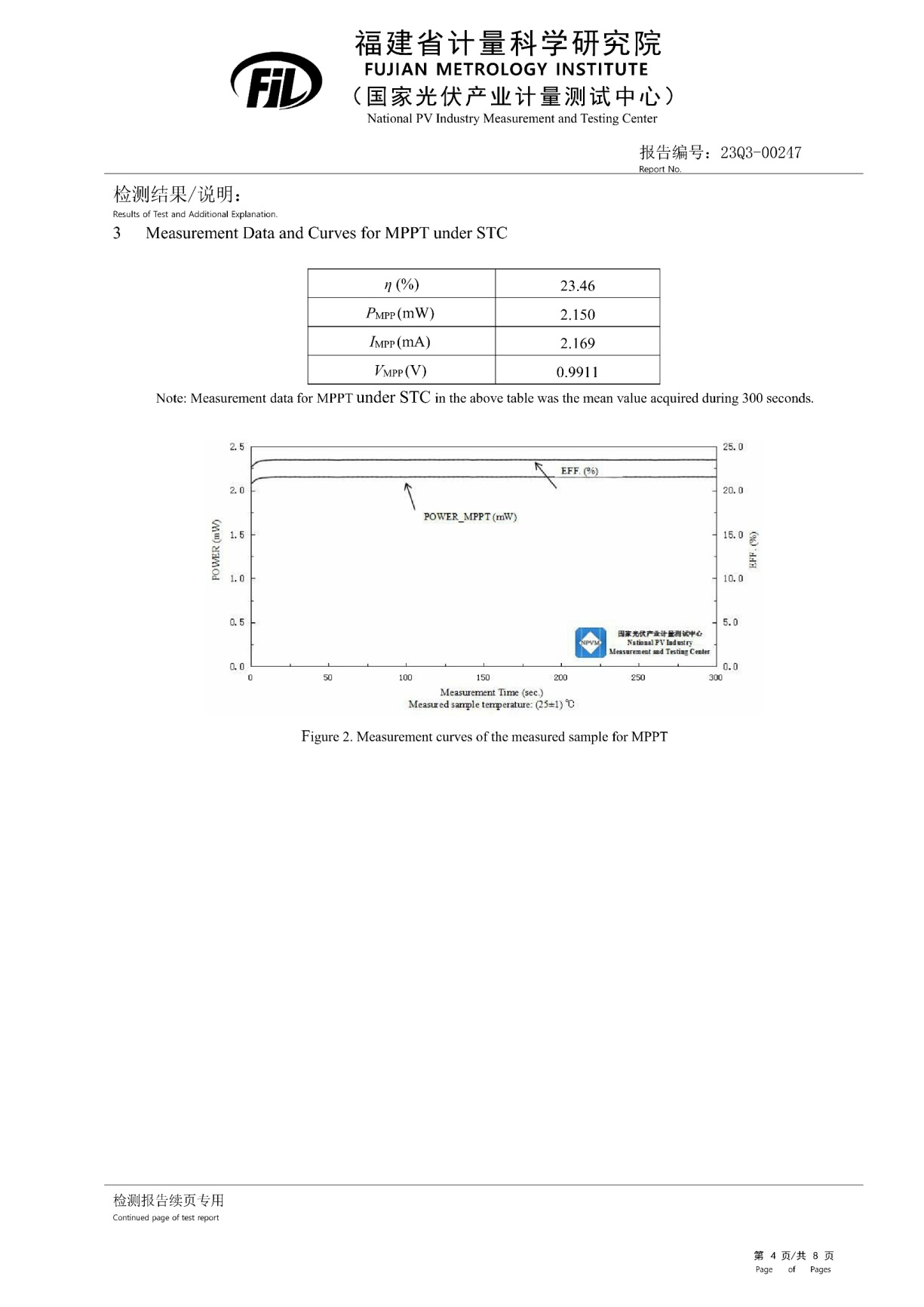


**Fig. S23** The certification report of the MPPT of the PSC of Fig. 4f determined by the Chinese national PV industry measurement and testing center

**Fig. S24** The PL spectra of the upper perovskite films deposited on glass without and with BM-TU modification

**Fig. S25** The *V*_OC_ as a function of light intensities for the control and modified PSCs

**Fig. S26** The design of the P1–P2–P3 pattern

**Fig. S27** The forward and reverse curve test results of the perovskite solar module

**Fig. S28** The thermal stability with and without BM-TU modified unencapsulated devices for 85 ℃


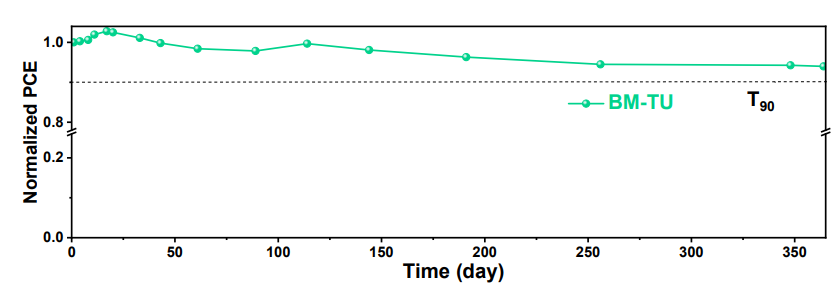


**Fig. S29** The storage stability for 60 PSCs

# Supplementary Tables

**Table S1** The binding energy between perovskite, Al_2_O_3_ and passivation atom was calculated by DFT

| **Sample** | **TU(S)** | **BM-TU(N)** | **BM-TU(S)** | **BM-TU(O)** |
| --- | --- | --- | --- | --- |
| FAPbI_3_ (Pb) | -1.125 | -1.968 | -2.056 | -1.380 |
| Al_2_O_3_(Al) | / | / | -1.987 | -1.568 |

**Table S2** TRPL fitting results by two-component exponential decay model

| **Sample** | **τ_ave_ / ns** | **τ_1_ / ns** | **τ_2_ / ns** | **A_1_** | **A_2_** | **I_0_** |
| --- | --- | --- | --- | --- | --- | --- |
| Control | 297.68 | 129.80 | 332.23 | 142.51 | 270.58 | 1.766 |
| BM-TU | 530.99 | 152.51 | 575.44 | 125.36 | 282.92 | 7.662 |

Calculation of *τ*_ave_: *τ*_ave_ = (A_1_*τ*_1_^2^+A_2_*τ*_2_^2^)/(A_1_*τ*_1_+A_2_*τ*_2_)

It is worth noting that the curves were fitted by two-component exponential decay equation $I(t)=I_{0}+A_{1}exp(-t/\tau_{1})+A_{2}exp(-t/\tau_{2})$and the fitted parameters are summarized in **Table S2**. Thereinto, the $\tau_{ave}$can be derived from the formula $\tau_{ave}=(A_{1}\tau_{1}^{2}+A_{2}\tau_{2}^{2})/(A_{1}\tau_{1}+A_{2}\tau_{2})$*, i*n which the$\tau_{1}$ and$\tau_{2}$ represent the fast and slow decay lifetime, respectively.

**Table S3** Summary of PCE for solar modules based on blade coating in recent years

| **Area [cm^2^]** | ***V*_OC_ [V]** | ***J*_SC_[mA cm^-2^]** | **FF [%]** | **PCE [%]** | **References** |
| --- | --- | --- | --- | --- | --- |
| 10 (Ap) | 6.870 | 3.55 | 76.90 | 18.80 | [S5] |
| 12.4 (Ap) | 5.948 | 5.01 | 77.55 | 23.09 | [S6] |
| 12.53 (Ap) | 7.140 | 3.98 | 78.90 | 22.40 | [S7] |
| 14.08 (Ap) | 7.700 | 2.98 | 71.00 | 16.37 | [S8] |
| 15.90 (Ap) | 7.520 | 2.70 | 73.03 | 14.82 | [S9] |
| 18.00 (Ap) | 5.360 | 4.17 | 63.20 | 14.13 | [S10] |
| 20.25 (Ap) | 7.000 | 3.01 | 73.10 | 15.28 | [S11] |
| 22.40 (Ap) | 8.130 | 3.16 | 80.00 | 21.40 | [S12] |
| 25.03(Ap) | 8.120 | 3.28 | 72.30 | 19.30 | [S13] |
| 29.54 (Ap) | 8.715 | 2.83 | 75.41 | 18.60 | [S14] |
| 35.80 (Ap) | 11.700 | 2.045 | 77.30 | 18.50 | [S15] |
| 37.83 (Ap) | 12.910 | 1.72 | 69.00 | 15.29 | [S8] |
| 44.40 (Ap) | 12.908 | 1.829 | 76.20 | 18.00 | [S14] |
| 49.60 (Ap) | 16.500 | 1.034 | 54.70 | 9.30 | [S16] |
| 53.60 (Ap) | 11.830 | 1.80 | 62.00 | 13.32 | [S17] |
| 60.84 (Ap) | 14.910 | 1.68 | 80.61 | 20.18 | This work |
| 63.70 (Ap) | 18.974 | 1.15 | 76.00 | 16.40 | [S18] |
| 66.00 (Ac) | 14.400 | 1.40 | 61.20 | 12.60 | [S19] |
| 81.00 (Ap) | 15.460 | 1.71 | 76.30 | 20.15 | [S20] |
| 205 (Ap) | 20.895 | 1.01 | 72.00 | 15.30 | [S12] |

**Supplementary References**

1. T. Xu, W. Xiang, J. Yang, D. J. Kubicki, W. Tress et al., Interface modification for efficient and stable inverted inorganic perovskite solar cells. Adv. Mater. **35**, 2303346 (2023). <https://doi.org/10.1002/adma.202303346>
2. H. Zhang, Q. Tian, W. Xiang, Y. Du, Z. Wang et al., Tailored cysteine-derived molecular structures toward efficient and stable inorganic perovskite solar cells. Adv. Mater. **35**, 2301140 (2023). <https://doi.org/10.1002/adma.202301140>
3. R. Xu, F. Pan, J. Chen, J. Li, Y. Yang et al., Optimizing the buried interface in flexible perovskite solar cells to achieve over 24% efficiency and long-term stability. Adv. Mater. **36**, 2308039 (2024). <https://doi.org/10.1002/adma.202308039>
4. R. He, W. Wang, Z. Yi, F. Lang, C. Chen et al., Improving interface quality for 1-cm^2^ all-perovskite tandem solar cells. Nature **618**, 80 (2023). <https://doi.org/10.1038/s41586-023-05992-y>
5. J. Zhao, S. O. Fürer, D. P. McMeekin, Q. Lin, P. Lv et al., Efficient and stable formamidinium-caesium perovskite solar cells and modules from lead acetate-based precursors. Energy Environ. Sci. **16**, 138-147 (2023). <https://doi.org/10.1039/D2EE01634F>
6. C. Huang, S. Tan, B. Yu, Y. Li, J. Shi et al., Meniscus-modulated blade coating enables high-quality α-phase formamidinium lead triiodide crystals and efficient perovskite minimodules. Joule (2024). <https://doi.org/10.1016/j.joule.2024.06.008>
7. T. Hou, M. Zhang, X. Sun, Y. Wang, K. Chen et al., Methylammonium-free ink for low-temperature crystallization of α-fapbi_3_ perovskite. Adv. Energy Mater. **14**, 2400932 (2024). <https://doi.org/10.1002/aenm.202400932>
8. Y. Yang, Y. Wang, Z. Qu, K. Zhang, T. Liang et al., Volatile dual-solvent assisted intermediate phase regulation for anti-solvent-free perovskite photovoltaics. Angew. Chem. Int. Ed. **62**, e202300971 (2023). <https://doi.org/10.1002/anie.202300971>
9. S. Siegrist, J. Kurisinkal Pious, H. Lai, R. K. Kothandaraman, J. Luo et al., Stabilizing solution-substrate interaction of perovskite ink on pedot: Pss for scalable blade coated narrow bandgap perovskite solar modules by gas quenching. Sol. RRL. **8**, 2400447 (2024). <https://doi.org/10.1002/solr.202400447>
10. J. Zeng, L. Bi, Y. Cheng, B. Xu, A. K.-Y. Jen. Self-assembled monolayer enabling improved buried interfaces in blade-coated perovskite solar cells for high efficiency and stability. Nano Res. Energy. **1**, e9120004 (2022). <https://doi.org/10.26599/NRE.2022.9120004>
11. S. Qiu, M. Majewski, L. Dong, D. Jang, V. M. L. Corre et al., In situ probing the crystallization kinetics in gas-quenching-assisted coating of perovskite films. Adv. Energy Mater. **14**, 2303210 (2024). <https://doi.org/10.1002/aenm.202303210>
12. T. Bu, L. K. Ono, J. Li, J. Su, G. Tong et al., Modulating crystal growth of formamidinium-caesium perovskites for over 200 cm^2^ photovoltaic sub-modules. Nat. Energy **7**, 528-536 (2022). <https://doi.org/10.1038/s41560-022-01039-0>
13. M. A. Uddin, P. J. S. Rana, Z. Ni, X. Dai, Z. Yu et al., Blading of conformal electron-transport layers in p-i-n perovskite solar cells. Adv. Mater. **34**, 2202954 (2022). <https://doi.org/10.1002/adma.202202954>
14. Y. Deng, S. Xu, S. Chen, X. Xiao, J. Zhao et al., Defect compensation in formamidinium-caesium perovskites for highly efficient solar mini-modules with improved photostability. Nat. Energy **6**, 633-641 (2021). <https://doi.org/10.1038/s41560-021-00831-8>
15. S. Chen, X. Xiao, H. Gu, J. Huang. Iodine reduction for reproducible and high-performance perovskite solar cells and modules. Sci. Adv. **7**, eabe8130 (2021). <https://doi.org/10.1126/sciadv.abe8130>
16. J. Küffner, T. Wahl, M. Schultes, J. Hanisch, J. Zillner et al., Nanoparticle wetting agent for gas stream-assisted blade-coated inverted perovskite solar cells and modules. ACS Appl. Mater. Interfaces **12**, 52678-52690 (2020). <https://doi.org/10.1021/acsami.0c15428>
17. J. Zhang, T. Bu, J. Li, H. Li, Y. Mo et al., Two-step sequential blade-coating of high quality perovskite layers for efficient solar cells and modules. J. Mater. Chem. A. **8**, 8447-8454 (2020). <https://doi.org/10.1039/D0TA02043E>
18. Y. Deng, C. H. Van Brackle, X. Dai, J. Zhao, B. Chen et al., Tailoring solvent coordination for high-speed, room-temperature blading of perovskite photovoltaic films. Sci. Adv. **5**, eaax7537 (2019). <https://doi.org/10.1126/sciadv.aax7537>
19. M. Ernst, J.-P. Herterich, C. Margenfeld, M. Kohlstädt, U. Würfel. Multilayer blade-coating fabrication of methylammonium-free perovskite photovoltaic modules with 66 cm^2^ active area. Sol. RRL **6**, 2100535 (2022). <https://doi.org/10.1002/solr.202100535>
20. J. Chung, S. W. Kim, Y. Li, T. Mariam, X. Wang et al., Engineering perovskite precursor inks for scalable production of high-efficiency perovskite photovoltaic modules. Adv. Energy Mater. **13**, 2300595 (2023). <https://doi.org/10.1002/aenm.202300595>
